# Optimizing Cardiac Wireless Implant Communication: A Feasibility Study on Selecting the Frequency and Matching Medium

**DOI:** 10.3390/s23073411

**Published:** 2023-03-24

**Authors:** Bilal Amin, Muhammad Riaz ur Rehman, Muhammad Farooq, Adnan Elahi, Kevin Donaghey, William Wijns, Atif Shahzad, Patricia Vazquez

**Affiliations:** 1 Smart Sensors Laboratory, College of Medicine, Nursing Health Sciences, University of Galway, H91 TK33 Galway, Ireland; 2 Electrical and Electronic Engineering, University of Galway, H91 TK33 Galway, Ireland; 3 Aurigen Medical, Atlantic Technological University (ATU) Innovation Hub, H91 FD73 Galway, Ireland; 4 Centre for Systems Modeling and Quantitative Biomedicine, University of Birmingham, Birmingham B15 2TT, UK

**Keywords:** cardiac care, cardiac wireless implantable medical devices, dielectric properties, electromagnetic waves, optimal frequency, implant antenna, penetration depth, transmission line

## Abstract

Cardiac wireless implantable medical devices (CWIMD) have brought a paradigm shift in monitoring and treating various cardiac conditions, including heart failure, arrhythmias, and hypertension. One of the key elements in CWIMD is the implant antenna which uses radio frequency (RF) technology to wirelessly communicate and transmit data to external devices. However, wireless communication with a deeply implanted antenna using RF can be challenging due to the significant loss of electromagnetic (EM) signal at the air–skin interface, and second, due to the propagation and reflection of EM waves from different tissue boundaries. The air–skin interface loss of the EM wave is pronounced due to the absence of a matching medium. This paper investigates the EM propagation losses in the human body and presents a choice of optimal frequency for the design of the cardiac implant antenna and the dielectric properties of the matching medium. First, the dielectric properties of all tissues present in the human thorax including skin, fat, muscle, cartilage, and heart are analyzed as a function of frequency to study the EM wave absorption at different frequencies. Second, the penetration of EM waves inside the biological tissues is analyzed as a function of frequency. Third, a transmission line (TL) formalism approach is adopted to examine the optimal frequency band for designing a cardiac implant antenna and the matching medium for the air–skin interface. Finally, experimental validation is performed at two ISM frequencies, 433 MHz and 915 MHz, selected from the optimal frequency band (0.4–1.5 GHz) suggested by our analytical investigation. For experimental validation, two off-the-shelf flexible dipole antennas operating at selected ISM frequencies were used. The numerical and experimental findings suggested that for the specific application of a cardiac implant with a penetration depth of 7–17 cm, the most effective frequency range for operation is within 0.4–1.5 GHz. The findings based on the dielectric properties of thorax tissues, the penetration depth of EM waves, and the optimal frequency band have provided valuable information on developing and optimizing CWIMDs for cardiac care applications.

## 1. Introduction

Cardiovascular diseases are the main cause of mortality worldwide, representing one-third of all global deaths [1]. Despite medical advances and general awareness of the associated risk factors, the prevalence of cardiovascular disorders keeps rising over time, almost doubling in value from 1990 to 2019 (271 million to 523 million) [2]. Common cardiovascular diseases are heart failure, heart valve disease, coronary heart disease, pericarditis, aortic aneurysm, cardiomyopathy, cardiac arrhythmia etc., although ischemic heart diseases are the most prevalent [3,4]. This represents a social and economic burden, with an estimated global cost of $555 billion in 2015, and is expected to be more than double by 2035 to $1.1 trillion [5]. Most of these resources are used in hospital inpatient care, followed by pharmacologic treatment [6], and represent a considerable part of the health care resources of all nations. The advent of cardiac implantable devices has increased the quality of life in patients, providing an independent lifestyle and longer life without recurrent hospitalization. As technology progresses in this field, the use of cardiac implantable electronic devices to control the cardiovascular disease is increasing [7,8,9,10,11]. Examples of such implantable devices include implantable cardioverter defibrillators, pacemakers, cardiac resynchronization therapy devices [12,13], and hemodynamic sensors [14].

Medical implants usually rely on RF signals to obtain power and communicate data to an external unit, as it is a well-established means of communication between two devices or multiple devices over the air/free space [8]. The range of communication depends on the communication channel properties and the frequency of the RF signal used for the communication. With different RF frequencies, the air/free space channel attenuates the RF signal, thus limiting the distance of propagation for the RF signal [9]. This attenuation effect is even more pronounced in the human body, as it presents higher losses to the RF signals as compared to the air/ free space [12]. The different tissues in the body offer characteristic impedance, conductivity, and dielectric constants for the incoming RF signal, and these parameters will dictate the degree of absorption generated [15]. In addition, the presence of different interfaces between tissue layers causes scattering, with consequent additional losses in the signal [9]. The attenuation of the RF signal can be observed linearly with the increase in the distance of communication [16]. However, the slope of the attenuation has a relation with the frequency of the RF signal. For a low-frequency RF signal, the slope of attenuation is lower than the high-frequency RF signal. As we increase the RF frequency, the slope of attenuation increases exponentially [16].

**Figure 1:**
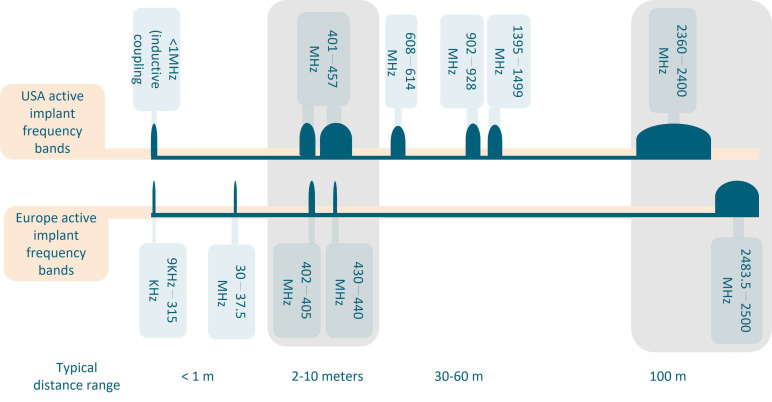
Frequency bands allocated to medical devices: an overview of the USA and European regulations, and the typical distance of these frequency bands. Common frequencies to both USA and European regulations are highlighted in grey.

The common frequency bands used for communication with the cardiac implants are Medical Implant Communication Services (MICS) 401–457 MHz range, Wireless Medical Telemetry Services (WMTS), with 608 to 614 MHz and the 1.4 GHz frequency band, and finally the more generical industrial, scientific, medical band (ISM), at 2.45 GHz [17]. Figure 1 gives an overview of the existing frequency bands allocated to medical devices in the USA and Europe. It can be observed that there are two overlapping frequency bands in both regulatory bodies, namely the MICS band and the ISM band (the latter, at 2.4 GHz).

Historically, the ISM band was the first to allocate its frequency band for medical devices (in 1947), but progress in RF technology promoted the expansion to the more specific MICS band, and later on the WMTS band [18]. The MICS range allows a more relaxed regulation of power requirements compared to the ISM. In addition, the former ensures low interferences coming from other equipment, which is not the case in the ISM band (as the frequency space is shared with non-specific short-range devices, such as Bluetooth, Wi-Fi, Zigbee, etc.) [17]. The MICS band is used for ultra-low power medical devices communication, requiring a reasonable antenna size and offering good signal propagation, due to small attenuation compared to higher frequencies [19]. However, it may not be suitable for applications where the location of the implant restricts the size of the implanted antenna. In addition, due to its low available bandwidth, only low-data rate applications favor the MICS band. By contrast, the ISM and ultrawideband (UWB) bands provide higher bandwidth and reduced antenna size, at the cost of poor propagation of the signal in the human body, and consequently with significant attenuation [20]. Based on the location of the implant in the body and functionalities supported by the cardiac implants, the operating frequency band and antenna size become critical design considerations to optimize the communication between the implants and the external reading device. In the available literature and commercial devices, most implants use 1.7 GHz ISM band, 2.4 GHz ISM band, or UWB for implant communication, despite the potential advantage of using lower frequencies to improve the link distance between the implanted and external antennas [19]. Lower RF attenuation in the MICS frequency band may therefore be an important factor for the improvement of communication stability in implanted cardiac systems since the site of implantation is deep and involves different tissue layers such as skin, muscle, and bone [20]. Signal losses are pronounced here due to the depth of the implantation (attenuation) and the different interfaces between tissues (scattering). Hence, it would be of value to perform a comparative analysis of signal propagation between frequencies in the high range of the ISM spectrum and the MICS band, as it would provide a quantifiable measurement of the effect of the frequency choice in the RF losses incurred [19]. Such a study could predict the range of distance in which the implantable system could provide stable communication between internal and external antennas. In [21], the ideal frequency for efficient RF power transfer through multiple layers of biological tissues was examined. The study utilized skin, fat, and muscle layers to model the tissues and found that frequencies greater than 1 GHz were optimal, especially when using a small receive coil located at a distance of up to 6 cm. However, this study does not provide any analysis for frequencies below 1 GHz. In [22], a study was conducted to determine the efficient frequency for deep implants located beyond 5 cm. This study considered the multi-layered composition of biological tissues, including skin, fat, muscle, bone, and heart. The results indicated that the optimal frequency for these deep implants was 1.6 GHz. In [23], a theoretical analysis was conducted on the optimal frequency for multi-layered tissues consisting of skin, fat, muscle, bone, and heart tissues. The study examined a coil-based source operating from a distance of 0.6 cm to 6 cm, over a frequency range of 100 MHz to 4 GHz. The results indicated that the optimal frequency for the specific source design was 2.6 GHz. However, the study did not analyze dipole antenna-based source designs beyond 6 cm. Meanwhile, the authors in [24] presented an RF channel modelling for cardiac implants, utilizing detailed numerical simulations over a frequency range of 300 MHz to 3 GHz. The study determined the optimal frequency for a cardiac implant to be between 2.4 GHz and 2.5 GHz due to design constraints. However, it is worth noting that the muscle model used in the numerical analysis consisted of only blood, muscle, and heart tissues. This study aimed to address the shortcomings of previous research by determining the optimal frequency range for cardiac implant wireless communication based on an analysis of the dielectric properties and penetration depths of human thorax tissues (skin, fat, muscle, cartilage, and heart) across a range of frequencies (0.4–3 GHz). The results indicated that varying frequencies exhibited different depths of tissue penetration, with lower frequencies being associated with greater penetration depths.

Therefore, first, this study has collated and analyzed the dielectric properties of various tissues inside the human thorax from the literature. The human thorax is mainly composed of skin, fat, muscle, cartilage, and heart. The dielectric properties play an important role in determining the propagation behavior of EM waves inside the tissues. These properties affect the attenuation and dispersion of the EM waves as a function of frequency [25,26]. An understanding of the dielectric properties of human thorax tissues will help to optimize the frequency and design of cardiac implant antennas for optimal signal transmission and minimize loss of power inside the human body [27]. Once the dielectric properties of thorax tissues were analyzed, the penetration depth of EM waves inside the thorax tissues was investigated. The penetration depth is an important factor to consider when designing a cardiac implant antenna, as it determines the distance at which the signal strength will be significantly reduced [28]. The penetration depth is also related to the frequency of the EM waves, as lower-frequency waves can penetrate deeper into the tissue than higher-frequency waves [15]. To analyze the optimal frequency band and matching medium dielectric properties, a transmission line (TL) formalism approach as the one adopted by Scapaticci et al. [29] for microwave imaging applied to cerebrovascular diseases was used for finding an optimal frequency band for cardiac implant antenna design and matching medium for reducing EM waves loss at air-skin interface. The optimum choice for the matching medium helps to improve the coupling between the incident wave and the tissues [30]. Further, to validate the numerical findings of the TL formalism approach, and to investigate the quality of the received signal for two different frequency bands, two sets of off-the-shelf antennas were used operating at two different ISM frequency bands (433 MHz and 915 MHz). The antennas were tested in a bio-phantom that emulated the characteristics of human blood. The bio-phantom was composed of Triton X-100, deionized water, and sodium chloride. Triton X-100-based bio-phantoms are a good choice for use in experiments because they have better heat stability than other materials, which allows for experiments to be conducted at both room and human body temperatures. Additionally, their dielectric properties remain stable for up to one year [31]. In summary, this study examined the dielectric properties of human thorax tissues, identified a suitable frequency band for designing antennas for cardiac implants, determined the appropriate matching medium for maximum EM field penetration in the heart, developed a bio-phantom to mimic human blood, and validated the chosen frequency band through experiments with two different antennas. The results of the study will aid in the development and improvement of antennas for use in cardiac care applications.

The remainder of this paper is structured as follows: Section 1 provides a literature review of CWIMDs, the cardiac implant antenna, and the impact of frequency and matching medium on propagation losses inside the human body. Section 2 discusses the methodology used to address the objectives of the study, including the dielectric properties of human thorax tissues, the penetration depth of EM waves inside the human thorax tissues, transmission line analysis for the choice of frequency and matching medium properties, preparation of bio-phantom, and experimental setup for investigating signal quality in two different frequency bands. Section 3 presents the results and discussion on the objectives of this study. Finally, Section 4 concludes and presents future work.

## 2. Materials and Methods

### 2.1. Dielectric Properties of Biological Tissues

Dielectric properties, namely relative permittivity and conductivity, can be used to describe the phenomena of electromagnetic (EM) wave reflection and propagation through biological tissues [32]. The dielectric properties of biological tissues are found to be frequency-dependent, due to varying levels of EM dispersion in the tissue [31]. Moreover, the dielectric properties also dictate the loss profile of EM waves inside the biological tissues. Therefore, the analysis of the dielectric properties of biological tissues is of paramount importance to understanding the reflection and propagation mechanism of different biological tissues and hence choosing an optimal frequency band for cardiac wireless communication [15,27,30]. To develop an anatomically realistic scenario for cardiac implant application, we have considered the human thorax as our target anatomical location. The human thorax was modelled as a lossy multi-layered medium consisting of five layers: skin, fat, muscle, cartilage, and heart. The dielectric measurement data of considered tissues was acquired from Gabriel et al. [33] for a frequency range of 0.4–2.5 GHz, as it is the most comprehensive study, widely used for characterizing the dielectric properties of biological tissues. The data acquired from Gabriel et al. [33] was plotted in MATLAB (The MathWorks, Natick, MA, USA).

### 2.2. Penetration Depth of EM Waves in Biological Tissues

The EM waves penetrate the biological tissues to varying depths, depending on the frequency and type of tissue [31]. In general, higher-frequency EM waves penetrate less deeply than lower-frequency EM waves. At frequencies below 100 MHz, EM waves penetrate most organic tissues to a depth of around 10 cm [27]. At higher frequencies, such as those used in medical imaging, penetration depths can range from a few mm to a few cm [34]. Moreover, the penetration depth for cardiac implant applications can vary depending on the implant type and the patient’s specific requirements. Generally, implants are placed between 0.5 and 3 cm below the skin, but some may be placed up to 5 cm below the skin [35]. Therefore, the analysis of the penetration depth of biological tissues is of paramount importance through different biological tissues and hence to choose an optimal frequency band for cardiac wireless communication. The penetration depth of EM waves in considered tissues is analyzed as a function of frequency. The penetration depth data of considered tissues were acquired from Gabriel et al. [36] for a frequency range of 0.4–2.5 GHz. Gabriel et al. [36] have presented the penetration depth of various biological tissues as a function of frequency. The data acquired from Gabriel et al. [36] was plotted in MATLAB (The MathWorks, Natick, MA, USA).

### 2.3. Choice of Frequency and the Matching Medium

The choice of frequency and relative permittivity of the matching medium for the cardiac implantable antenna is performed by adopting the TL formalism approach as proposed by Scapaticci et al. [29]. The frequency and the relative permittivity of the matching medium define the degrees of freedom of the proposed system; therefore, their validity and ease of use should be ensured [29]. The choice of these parameters should be performed to meet the following two objectives:

1. The first objective is to transfer the maximum power of the incident EM wave to the target tissue, which is the heart in our case;
2. The second objective is to achieve the maximum spatial resolution, which is dictated by the wavelength in the matching medium, i.e., the medium where the transmitting/receiving antennas are located [29].

In addition, the choice of the relative permittivity of the matching medium determines the EM wave penetration into the medium under investigation; the higher the matching between electrical discontinuities, the higher the EM penetration would be [37]. To address this, a planar layered model was investigated as proposed by Scapaticci et al. [29]. The planar layered model considers the TL formalism approach and is therefore convenient for a first-order analysis [28]. The TL formalism approach helps to identify the optimal frequency band based on the transmission coefficient (T); moreover, it helps to identify the relative permittivity of the matching medium. In the TL formalism approach, the anatomical site to be investigated is modelled as a one-dimensional (1-D) planar layered model, where each layer is assigned an equivalent impedance (Z) [29]. The penetration of EM waves into the heart can be assessed from the strength of the transmission coefficient. Moreover, the choice of matching medium’s relative permittivity is dictated based on the optimal frequency band.

#### 2.3.1. Planar Layered Model

**Figure 2:**
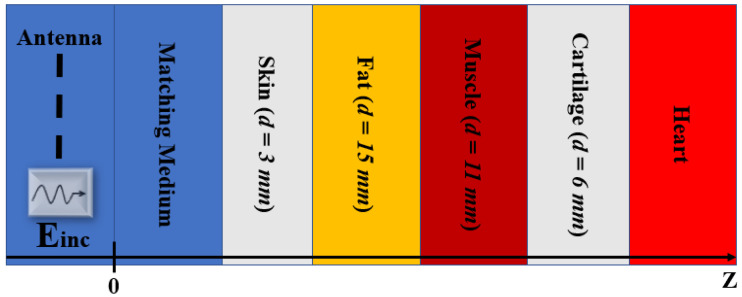
The 1-D planar layered model of the human thorax. The model is composed of five layers including skin, fat, muscle, cartilage, and heart. E_inc_ is the probing wave. The different colours of the layers are chosen to distinguish between the considered tissues.

In the planar layered model, we have modelled the interaction of the probing wave (E_inc_) with the heart as a normal incidence of a plane wave on the 1-D multi-layered structure. The multi-layered structure including the matching layer, skin, fat, muscle, cartilage, and heart is shown in Figure 2. In the present case of a five-layered model starting from the matching medium, the thickness of the first layer of skin is assumed to be 3 mm, the second layer of fat has a thickness of 15 mm, the muscle has a thickness of 11 mm, and cartilage has a thickness of 6 mm. The heart is modelled as a half region to guarantee maximum penetration of the EM field. The 1-D numerical modelling and finite difference time domain (FDTD) simulations were performed with MATLAB (The MathWorks, Natick, MA, USA).

**Figure 3:**
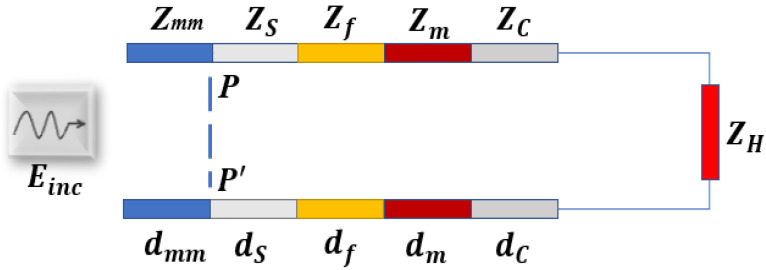
The transmission line model of 1-D thorax structure. The *Z_mm_*, *Z_s_*, *Z_f_*, *Z_m_*, *Z_C_*, and *Z_H_* represent the impedance of matching medium, plane PP′, skin, fat, muscle, cartilage, and heart respectively. The different colours of the layers are chosen to distinguish between the considered tissues.

The transmission line (TL) formalism approach analyzes the propagation of EM waves across a layered structure by modelling the tissue layers as transmission lines, with each tissue layer given a unique characteristic impedance (*Z_LM_*). In this study, the skin, fat, muscle, cartilage, and heart tissue layers are modelled using the corresponding impedances *Z_s_*, *Z_f_*, *Z_m_*, *Z_C_*, and *Z_H_* respectively. Additionally, as illustrated in Figure 3, the matching medium between the skin and the external antenna is modelled as *Z_mm_*. According to the impedance transfer equation, the impedance at any interface of the TL closed on a load can be evaluated by using the following equation:

(1) $$Z_{IF}=Z_{LM}\frac{Z_{LD}+jZ_{LM}tan(k_{LM}l_{LM})}{Z_{LM}+jZ_{LD}tan(k_{LM}l_{LM})};$$

where $Z_{IF}$ denotes the impedance at the interface of interest, $Z_{LD}$ denotes the impedance of the load, $Z_{LM}$ denotes the characteristic (or intrinsic) impedance of a specific tissue layer. The characteristic impedance of any specific tissue layer is modelled as:

(2) $$Z_{LM}=\sqrt{\frac{\mu_{o}}{\varepsilon_{o}\varepsilon_{tissue}}}$$

where *ε_tissue_* denotes the relative permittivity of each tissue layer under consideration, *μ_o_* and *ε_o_* denote permeability and relative permittivity of free space respectively. The wavenumber of a specific tissue layer ($k_{LM}$) can be defined as:

(3) $$k_{LM}=\omega \sqrt{\mu_{o}\varepsilon_{tissue}}$$

where ω is the angular frequency. The length of each tissue layer in TL formalism is chosen based on the thickness of each layer. The single-pole Cole-Cole model is used to simulate the dielectric characteristics of each tissue layer to account for the frequency dispersive behavior of biological tissues. The single-pole Cole-Cole model is expressed as:

(4) $$\varepsilon \left(\omega \right)=\varepsilon_{\infty }+\frac{∆\varepsilon }{1+j\omega \tau^{1-\alpha }}+\frac{\sigma_{i}}{j\omega \varepsilon_{o}}$$

where *ε_∞_* is the highest frequency permittivity, ∆ε is the change in the permittivity, *σ_i_* is the static ionic conductivity, τ is the relaxation constant, and α is the empirical parameter to broaden the dispersion. The values of each of these parameters were taken from Gabriel et al. [33] and are tabulated in Table 1.

In the TL formalism approach, the amount of incident power that is captured by the heart can be modelled using the transmission coefficient (*T*) and can be given as:

(5) $$T=1-{\left|\Gamma \right|}^{2}$$

where *Γ* denotes the reflection coefficient between the matching medium and the skin. The reflection coefficient can be given as:

(6) $$\Gamma =\frac{Z_{{PP}^{\prime }}-Z_{mm}}{Z_{{PP}^{\prime }}+Z_{mm}}$$

where *Z_mm_* denotes the impedance of the matching medium and *Z_PP_* denotes the equivalent impedance of the thorax. 

**Figure 4:**
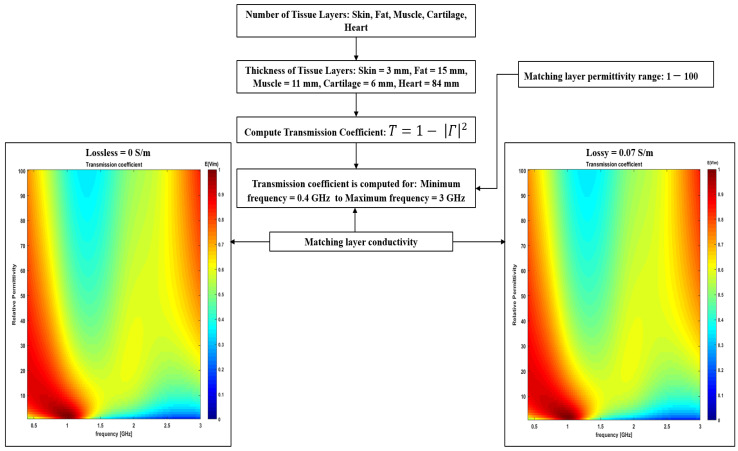
A detailed block diagram of the simulation model.

To analyze the role of coupling medium and choice of frequency, we have analyzed the transmission coefficient (T) as a function of frequency (400 MHz–3 GHz). The magnitude of the matching medium’s relative permittivity varied between 1 and 100; however, the matching medium was assumed lossless. The detailed block diagram of the simulation model is shown in Figure 4.

### 2.4. Experimental Setup for Distance and Signal Quality Measurement for Cardiac Implants

**Figure 5:**
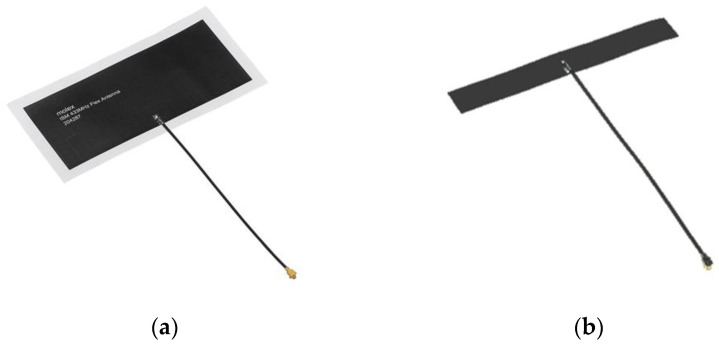
Antennas used for evaluating 433 MHz and 915 MHz ISM bands, with (**a**) ISM 433 MHz Flex Antenna and (**b**) ISM 868/915 MHz Antenna.

To analyze the signal quality measurement for cardiac implants, two antennas from Molex^®^ (Molex LLC, Lisle, IL, USA) were chosen for the communication performance evaluation of the 433 MHz and 915 MHz bands. The first is the ISM 433 MHz Flex Antenna (Figure 5a), which is a flexible antenna designed for the ISM band and has a linear polarization and omnidirectional radiation pattern. It has a length of 90 mm, a width of 40 mm, and a thickness of 0.1 mm. The second antenna is the ISM 868/915 MHz Antenna (Figure 5b), which is also a flexible antenna designed for the 868/915 MHz ISM band and has a linear polarization and omnidirectional radiation pattern. This antenna has a length of 79 mm, a width of 10 mm, and a thickness of 0.1 mm. Both antennas have a cable length of 100 mm and were attached to a backplane for ease of handling and precise positioning during the experiments.

**Figure 6:**
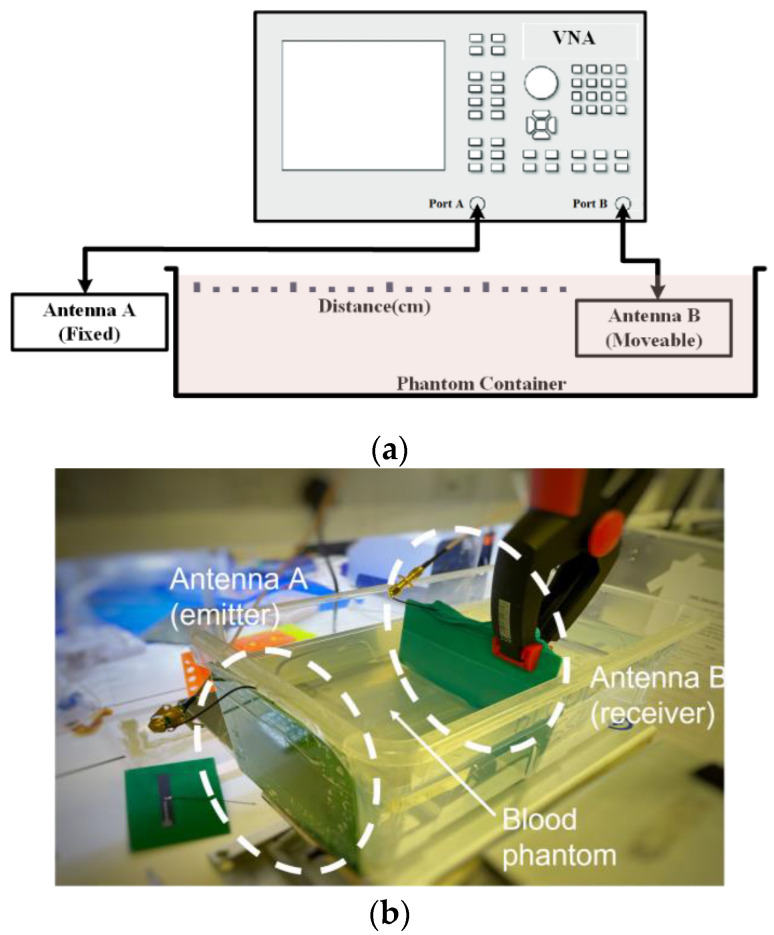
Experimental set-up for testing of communication bands; (**a**) schematic, (**b**) image of test-bench. Two antennas of the same frequency are connected to the VNA, with Antenna A outside the container. Antenna B is insulated with a latex glove and immersed in the blood phantom.

The experimental setup for the testing of communication bands is shown in Figure 6. A Keysight E5063A vector network analyzer (VNA) (Keysight Technologies, Santa Rosa, CA, USA) was used to measure the communication loss between the antennas. Two antennas of the same frequency are used for each set of experiments. An antenna A is taped to the outside of the container holding a blood phantom (acts as a transmitter). The antenna B (acts as receiver) is insulated with a latex glove and immersed in the liquid phantom in the container. Antenna A is fixed while antenna B is moved to a calibrated distance between antenna A and B. The reflection and transmission parameters (S_11_, S_22_ and S_12_) are measured for both communication bands 433 MHz and 915 MHz. The container has a dimension of 30 × 17 × 11 cm (length × width × height), with the phantom filling the container to a height of 7.5 cm (the plastic material has a thickness of 1.5 mm). This means that the phantom had an approximate volume of 3645 cm^3^.

### 2.5. Liquid Bio-Phantom Preparation

In this study, we have developed a liquid bio-phantom for blood. The bio-phantom material used is a mixture of Triton X-100 (Thermo Fisher Scientific Inc., Waltham, MA, USA), deionized water, and sodium chloride [38]. The Triton X-100-based liquid bio-phantoms mimic a variety of biological tissues such as the breast, bone, and head tissues [31,38]. The bio-phantoms were prepared based on the guidelines outlined by Nadine et al. [38]. The solution of Triton X-100, deionized water, and sodium chloride was added to a glass beaker and thoroughly mixed until the disappearance of air bubbles. Once the air bubbles were settled, the permittivity and conductivity of the bio-phantom were measured by employing an open-ended coaxial probe (OECL) technique. The composition of ingredients was adjusted until the dielectric properties of bio-phantom were close to the reference values. The measurements were recorded at the frequency of interest (0.5–8.5) GHz over 101 linearly spaced frequency points. The dielectric measurements were performed by Keysight slim form probe 85070E (Keysight Technologies, Santa Rosa, CA, USA) connected directly to the Keysight E5063A VNA [32]. The VNA was used to determine the reflection coefficient (S_11_) at 101 evenly spaced frequencies between 0.5 and 8.5 GHz, and a software program (Keysight N1500A) was utilized to convert the S_11_ measurements into the real and imaginary components of complex permittivity. Different quantities of Triton X-100, deionized water and sodium chloride were mixed to produce solutions with a wide variety of dielectric properties. Larger salt concentrations were utilized to increase conductivity, whereas higher Triton X-100 concentrations were employed to decrease the conductivity and permittivity of the solution [39].

## 3. Results and Discussion

This section first presents an analysis of the dielectric properties of the considered tissues. Second, the penetration depth of EM waves was analyzed for the considered tissues and a TL formalism approach is presented to investigate the choice of the optimal frequency band and dielectric properties of matching medium for cardiac antenna design. Finally, the experimental results with a bio-phantom, along with the interrogation of maximum achievable distance and signal quality between two sets of off-the-shelf antennas for two representative frequency bands are discussed. These frequencies were chosen as low and high values within the frequency band that this work predicts as optimal, and which are also allocated for medical device use.

### 3.1. Dielectric Properties of Biological Tissues

**Figure 7:**
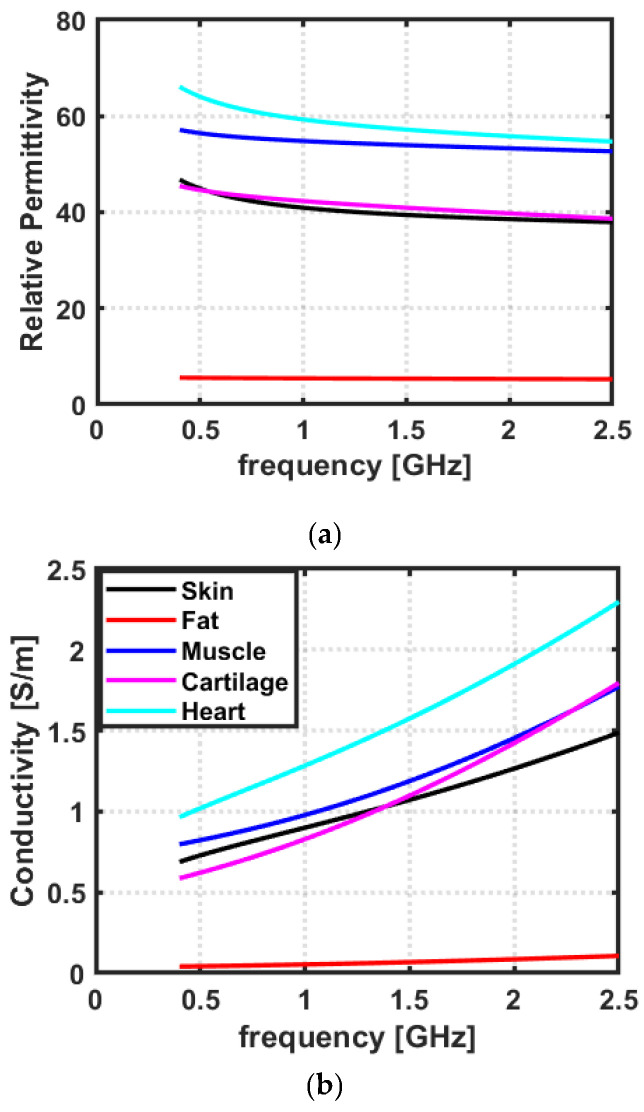
The (**a**) relative permittivity and (**b**) conductivity of the considered tissues as a function of frequency. The values are taken from Gabriel et al. [33].

The dielectric properties of biological tissues refer to the way tissues interact and respond to EM fields [40]. Moreover, the dielectric properties of biological tissues are important in several applications involving cardiac implants, such as pacemakers, defibrillators, and other devices that use electrical signals to regulate or stimulate the heart [41]. The dielectric properties of the tissue surrounding an implanted antenna can affect the performance and reliability of the antenna by influencing the way that EM waves propagate through the tissue. If the tissue has high dielectric losses, it can absorb a significant amount of the energy from the EM waves, which can reduce the effectiveness of the antenna [42]. On the other hand, if the tissue has low dielectric losses, it can allow the electrical signals to pass through more efficiently, which can improve the performance of the antenna [27]. Therefore, understanding and optimizing the dielectric properties of the tissues can be important in designing and implementing effective and reliable cardiac implant systems. To this end, this study has analyzed the dielectric properties of tissues present in the human thorax including skin, fat, muscle, cartilage, and heart. Figure 7a,b represent the relative permittivity and conductivity of all tissues present in the human thorax as a function of frequency. In this study, a frequency band of 0.4–2.5 GHz has been observed for cardiac implant application, as these frequencies are associated with medical applications due to their accessibility and associated wave propagation characteristics [17]. The dielectric profile of tissues suggests that a significant amount of contrast exists in terms of relative permittivity and conductivity among all tissues of the thorax. Further, it can be observed from Figure 7b, that the conductivity which represents the loss of EM waves inside the biological tissues increases as a function of frequency. Therefore, considering higher frequencies for cardiac implant applications will offer more loss to EM waves compared to the losses observed at the low-frequency band. 

### 3.2. Penetration Depth of EM Waves in Biological Tissues

**Figure 8:**
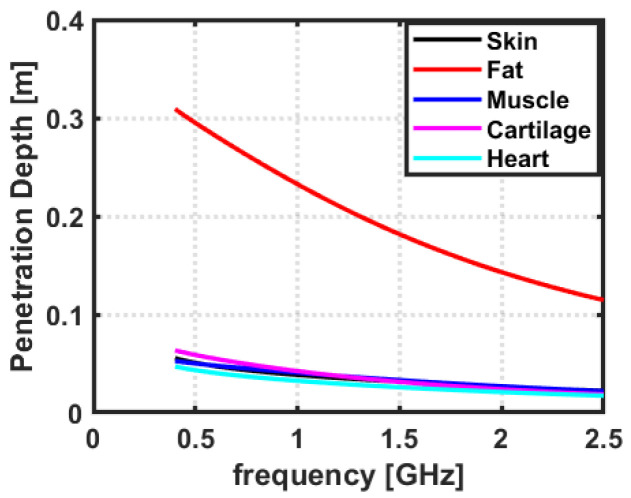
The penetration depth of considered tissues as a function of frequency. The values are taken from Gabriel et al. [36].

The penetration depth of EM waves in biological tissues determines how deeply the waves can penetrate the body [29,42]. This is important because, if the penetration depth is too shallow, the EM waves from an external source may not be able to reach the implant antenna for communication or powering the implanted sensor inside the heart, which could affect its function. Moreover, if the EM waves can penetrate too deeply into the body, they may interact with other tissues or organs in unintended ways, which could potentially cause harm. Therefore, choosing an optimum frequency band is very important for designing a reliable cardiac implant system. To analyze this fact and to find the optimal frequency range based on penetration depth we acquired data from Gabriel et al. [36] for the considered tissues present in the human thorax. Figure 8 represents the penetration depth of EM waves for the observed frequency band. It can be observed from Figure 8 that the EM wave penetration decreases as the frequency increases. Moreover, it can be observed that the heart tissue has the minimum penetration depth of EM waves in all considered tissues. Therefore, considering higher frequencies for antenna design would not be practicable for cardiac implant applications due to the less penetration depth of EM waves inside the heart.

### 3.3. Choice of Frequency and the Matching Medium (Transmission Line Analysis)

**Figure 9:**
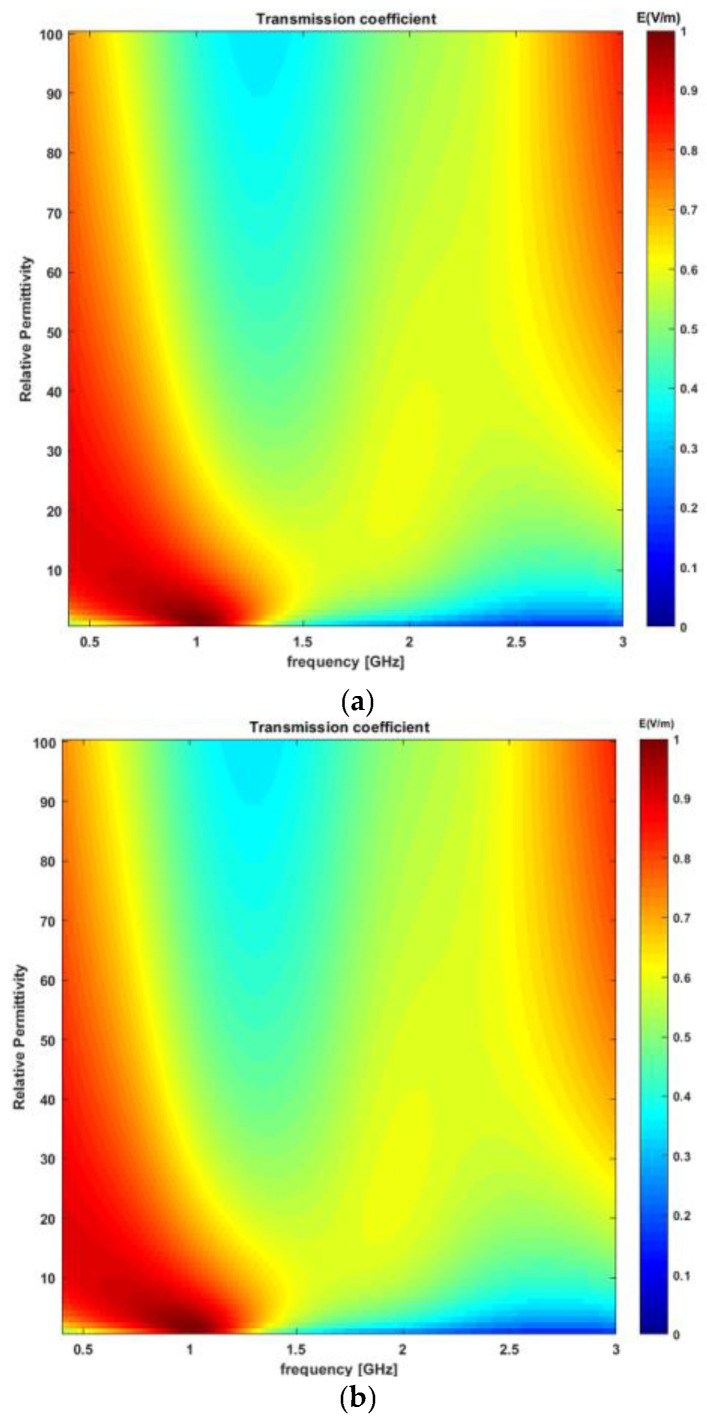
The transmission coefficient as a function of frequency and relative permittivity of matching medium for (**a**) lossless matching medium; (**b**) lossy matching medium with conductivity 0.07 S/m.

The transmission coefficient is assessed to determine the optimal frequency band and relative permittivity of the matching medium as a function of frequency (0.4–3 GHz), as shown in Figure 9a. The transmission coefficient was determined at the interface between the matching medium and the layered structure providing a simplified model of the thorax. The detailed TL formalism simulation model has been discussed in Section 2.3.1. In this study, the thickness of the matching layer is not evaluated, as the TL formalism approach does not account for the thickness of the matching layer. Instead, it only provides guidance on the choice of relative permittivity of the matching medium based on the feasible frequency band. The relative permittivity of the matching media was varied from 1 to 100; however, it was assumed that the matching medium was lossless. Figure 9a shows the transmission coefficient as a function of frequency and relative permittivity of the matching medium. It can be observed from Figure 9a that there exists a “forbidden transmission frequency band” between 1.5 and 2.8 GHz where the magnitude of the transmission coefficient is less than 0.6. Consequently, less power is delivered to the target between 1.5 and 2.8 GHz as compared to the other frequency bands. Therefore, the operating circumstances for the cardiac implant antenna for this frequency band do not appear to be favorable. The forbidden transmission frequency band arises because a noticeable difference exists in terms of the dielectric properties of each layer involved in the five-layer model. In addition to this, the electrical length of the low permittivity tissue layer such as fat which is sandwiched between two high dielectric properties medium, skin and muscle, which causes a strong mismatch and behaves like a waveguide at certain frequencies [30,43]. Further, it can be observed from Figure 9a that the magnitude of the transmission coefficient is strong around 3 GHz. However, the low penetration depth of the inspected tissues beyond 2.5 GHz, as shown in Figure 8, makes it impossible to accurately measure the useful signals beyond 2.5 GHz. Therefore, frequency bands beyond 2.5 GHz are less favorable for cardiac implant application. Taking all these considerations we concluded that a frequency band in the range of 0.4–1.5 GHz would be the most appropriate frequency band for cardiac implant application. Regarding the choice of relative permittivity of the matching medium, it can be observed from Figure 9a that any relative permittivity can be arbitrarily chosen between the frequency range of 0.4–1.5 GHz. Since the achievable spatial resolution depends upon the background’s medium wavelength, therefore, a matching medium having a large value of relative permittivity will be preferable [37]. However, higher relative permittivity values show degraded performances at the lower frequencies. Moreover, the choice of matching medium primarily depends upon factors such as conductive loss, relative permittivity, antenna matching, fabrication complexity, and ease of use [15]. One of the commonly used matching medium layers in RF, microwave and optical applications is λ/4 also known as the quarter-wave matching layer [44]. While λ/4 matching layers are a useful and commonly used technique, however, they do have some limitations including frequency dependence, temperature sensitivity and material selection. This study has proposed a guideline for selecting the relative permittivity of the matching medium based on the TL formalism approach [29]. Once the relative permittivity of the matching medium is determined, liquid/gel-based mimicking mixtures including oil-in-gelatine, Triton X-100 mixtures, and oil/water emulsion can be developed to mimic the matching medium [30]. In this study, a matching medium having a relative permittivity of 23 and a conductivity of 0.07 S/m has been proposed for cardiac wireless implant communication. Therefore, an oil/water emulsion can be prepared to achieve a conductivity of 0.05 S/m and relative permittivity of 23 [30]. Other fluids including safflower oil, glycerine, and acetone can also be used as a matching medium to achieve similar relative permittivity and conductivity. This study has only proposed the dielectric properties of the matching medium that can be used to minimize the EM signal loss at the air-skin interface for the proposed frequency band of (0.4–1.5 GHz) for cardiac wireless implant communication. Moreover, this study has not evaluated the effect of different thicknesses of the matching medium layer on the power transferred to the tissues. However, in general, the thicker the matching layer, the less power reaches the tissues. This is because the matching layer acts as an impedance-matching interface between the high-impedance transducer and the low-impedance tissues. As the thickness of the matching layer increases, the impedance mismatch becomes greater, resulting in a greater amount of energy being reflected toward the transducer instead of being transmitted into the tissues. This reflection can reduce the amount of energy available to penetrate the tissues, leading to reduced power to the target tissue. Therefore, a thin layer of the matching medium should be used at the air–skin interface.

To account for a more realistic scenario, the transmission coefficient was recalculated taking into account a lossy matching medium and evaluating its impact on the previous results. To this end, an oil/water emulsion was considered as the matching medium having a relative permittivity of 23 and a conductivity of 0.07 S/m [30]. The transmission coefficient was recalculated as a function of frequency (0.4–3 GHz), as shown in Figure 9b. The relative permittivity of the matching medium varied between 1 and 100. It can be observed from Figure 9b that no significant difference exists in the above findings when the matching medium was assumed lossless.

### 3.4. Liquid Bio-Phantom

To assess the validity of numerical outcomes of optimal frequency band through TL analysis, this study has performed an experimental investigation. To this end, a blood bio-phantom was developed to emulate the characteristics of the body and to test two sets of antennas resonating at two different frequency bands, 433 MHz and 915 MHz. The bio-phantom mixture was composed of Triton X-100, de-ionized water, and sodium chloride. The recipe for blood bio-phantom was obtained from Nadine et al. [38]. The composition of the ingredients of the bio-phantom is tabulated in Table 2. The bio-phantom mixture was highly viscous, however, due to the thorough mixing of the ingredients the bio-phantom was fully homogenous.

**Figure 10:**
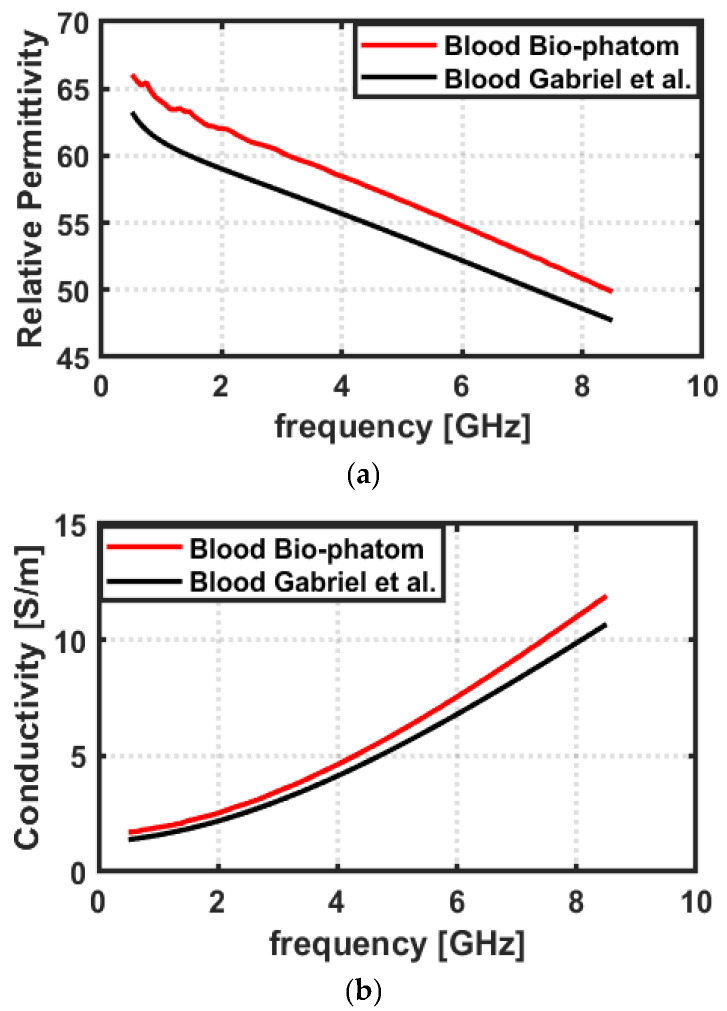
Dielectric properties of blood bio-phantom over 0.5–8.5 GHz frequency band: (**a**) Relative permittivity, (**b**) conductivity. The measured dielectric data of bio-phantom(red curve) is compared with the reference data (black curve) from Gabriel et al. [33].

The dielectric properties of the bio-phantom were measured using a Keysight slim form probe 85070E. The results of the measurements are shown in Figure 10, where the red curve represents the mean of five measurements taken at 101 evenly spaced frequencies between 0.5 and 8.5 GHz and the black curve represents reference data taken from Gabriel et al. [33]. The measurements were taken at multiple sites within the bio-phantom to ensure the homogeneity of the developed bio-phantom. The results show that the dielectric properties of the bio-phantom are well-aligned with the reference data and the average difference between the relative permittivity of the reference data and the proposed bio-phantom is less than 10%. The variations observed are in line with the literature on bio-phantoms for human biological tissues [31,38,42,45,46,47].

### 3.5. Experimental Findings for Distance and Signal Quality Measurement for Cardiac Implants

The measurement of reflection and transmission coefficients between a pair of antennas provides very useful information on the losses occurring during the propagation of the RF signal from the transmitting antenna (external to the blood bio-phantom) and the receiving antenna (inside the blood bio-phantom), which is immersed in the bio-phantom. From the analysis of parameters S_11_ it is possible to observe the resonance frequency of each antenna for this setup. The resonance frequencies of the antennas are shown in Table 3.

**Figure 11:**
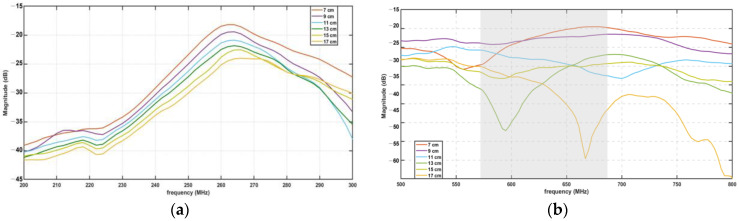
S_12_ parameters of (**a**) 433 MHz antenna and (**b**) 915 MHz antenna measured at different distances from the transmitting antenna (7 to 17 cm).

The S_12_ parameter, also known as the transmission coefficient, represents the power transferred from one antenna (in this case, the antenna connected to Port 2 of the VNA) to another antenna (connected to Port 1, outside the phantom). Figure 11 shows the variation of the S_12_ parameter at different distances (from 7 to 17 cm) between the two antennas. This can be used to study the effect of the distance between the two antennas on the power transfer between them. Additionally, it can be used to study the effect of the phantom on the coupling between the two antennas, as the distance increases the coupling between the two antennas will be reduced. Moreover, it can be observed from Figure 11 that the maximum values of S_12_ presented in the curves of the S_12_ parameter appear in the range delimited by the resonance frequency of the two antennas used in the experiment. The 433 MHz antennas show a maximum value of S_12_ around 264 MHz, and the 915 MHz antennas present their maximum around the 700 MHz value (although this set of curves presents a wider variation in the position of their maximum and a much wider bandwidth). The shift in the resonance frequencies of both sets of antennas is mainly observed because antenna B was placed inside the bio-phantom. The bio-phantom can change the effective permittivity and permeability of the antenna, which can affect the antenna’s resonance frequency. Additionally, when the antenna is placed inside the bio-phantom, it can experience additional losses, which can also shift the resonance frequency to lower values.

**Figure 12:**
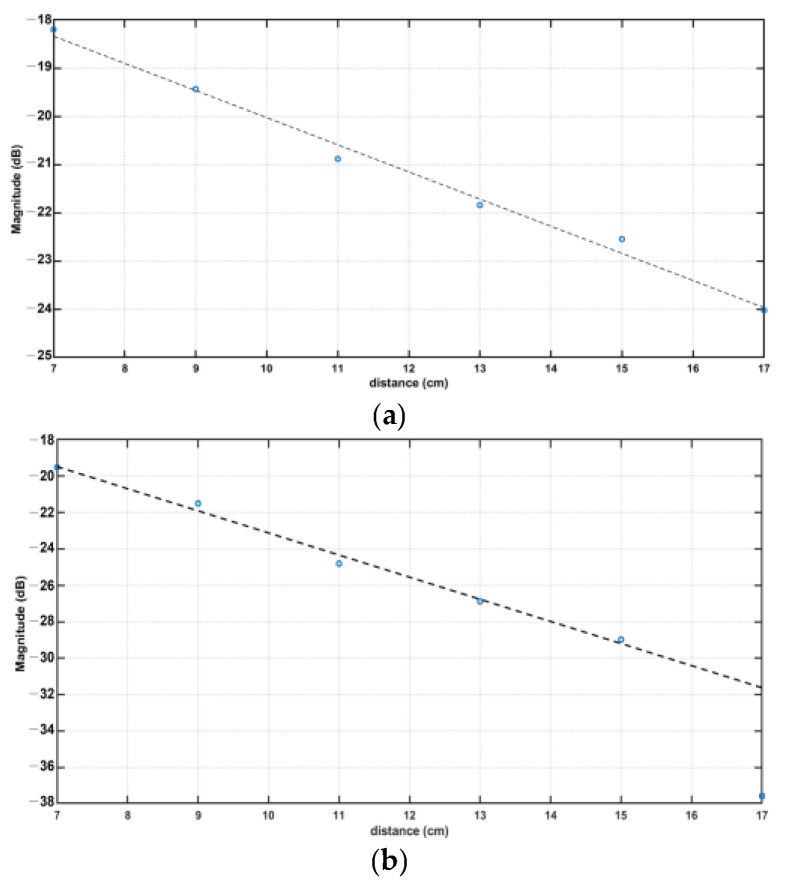
Maximum values of S_12_ parameters were measured at distances 7–17 cm, (**a**) a 433 MHz antenna (**b**) a 915 MHz antenna. The linear regression of these data presents slopes of −0.57 (R^2^ = 0.99) and −1.21 (R^2^ = 0.99), respectively.

The plots of the maximums found in the S_12_ parameter curves from Figure 11 are shown in Figure 12. Both frequencies under study show a linear decrease in their signal amplitude, except in the case of the 915 MHz, where distances higher than 15 cm present exponential losses. The 433 MHz graph shows a slope of −0.57, whereas the 915 MHz counterpart presented a slope of −1.21 (R^2^ = 0.99).

The linear regression of the maximums found on the S_12_ parameters of the antennas shows that the 433 MHz antenna has fewer propagation losses than the 915 MHz antenna, confirming the theoretical results that predicted the 433 MHz antenna performing better at transferring power to the receiving antenna. Additionally, if the 915 MHz antenna reveals more than double transmission losses than the 433 MHz antenna, it may indicate that the 915 MHz antenna is less efficient at transmitting power and may have more absorption or scattering losses in the bio-phantom. In addition, the higher frequency shows a non-linear decay at distances starting at 14 cm (Figure 12b), where maximums present similar values that are confounded with the noise floor of the equipment. Future studies should consider the comparison of S_12_ parameters of the antennas between the feasible frequency band (0.4–1.5 GHz) and the forbidden transmission frequency band (1.5–2.8 GHz). Such a comparison will further provide insight into the performance of the antennas in the feasible frequency band and their ability to transmit less power to the target in the forbidden transmission frequency band.

## 4. Conclusions

The choice of optimal frequency for a cardiac implant antenna provides the right balance of penetration depth, matching medium properties between the air–skin interface, and minimal interference with other devices so that it can provide a stable and maximum link distance to an external antenna. This is of particular relevance in implantable cardiac devices, as the depth of implantation is significant (an average of 10 to 15 cm) and requires the electromagnetic signal to travel through several tissue layers, incurring signal scattering and attenuation. This study proposed an optimal frequency band for designing such an antenna by investigating the dielectric properties and penetration depth of human thorax tissues (skin, fat, muscle, cartilage, and heart) as a function of frequency. The findings show clearly that different frequencies have different penetration depths in tissues, and confirm that lower frequencies tend to penetrate deeper. The study also adopted the TL formalism approach to finding the optimal selection of frequency and matching medium to maximize EM field penetration into the heart. The analysis suggested that a 0.4–1.5 GHz frequency band is the most suitable for designing a cardiac implant antenna, and a matching medium with relative permittivity between 10 and 30 is best for the air–skin interface. Finally, to investigate the numerical simulations, an experimental investigation was performed by utilizing 433 MHz and 915 MHz off-the-shelf antennas. These commercial antennas were selected to avoid variations in the manual prototyping of antennas. Furthermore, they use the same materials and configurations as similar as it is possible for an unbiased comparison of frequencies in the blood phantom. Although these antennas are optimized for free space, their detuning in the blood phantom should not affect the results regarding transmission losses as a function of distance, since the comparison between antennas of 433 MHz and 915 MHz was based on the slopes from their maximum values of S_12_ parameters at different distances between external and implanted antennas. This study used a blood phantom, as blood presents maximum losses due to its highest conductivity coefficient. The linear regression of the maximums found on the S_12_ parameters of both antennas shows that the 433 MHz antenna has fewer propagation losses than the 915 MHz antenna, with the latter revealing more than double transmission losses. For heart implant applications, choosing an antenna with minimal losses is fundamental to obtaining a stable link between the implant and the external antenna. Therefore, the allocated 401–450 MHz band for active medical devices would be the ideal frequency band for implantable cardiac devices. 

The proposed guidelines for the choice of optimal frequency band and matching medium properties would help in optimizing CWIMDs, leading toward the improvement of link quality and distance. Future work will focus on developing a miniaturized antenna for the proposed frequency band, allowing for smaller and more comfortable CWIMDs that can be implanted in the body with minimal invasiveness. Additionally, advanced materials such as metamaterials will be explored to improve the performance of the antenna, by increasing the efficiency of energy transfer and improving the radiation pattern.

## Figures and Tables

**Table 1:** Cole-Cole parameters of skin, fat, muscle, cartilage, and heart.

Tissue	$\varepsilon_{\infty }$	∆ε	τ	α	$\sigma_{i}$
Dry Skin	4	32	7.23 × 10^−12^	0	0.0002
Fat	2.5	3.0	7.96 × 10^−12^	0.2	0.01
Muscle	4	50	7.23 × 10^−12^	0.10	0.20
Cartilage	4.34	35.6	12.8 × 10^−12^	0.25	0.07
Heart	4	50.0	7.96 × 10^−12^	0.10	0.05

**Table 2:** Ingredients and quantities for bio-phantom material.

Bio-Phantom	Triton X-100 (Vol %)	De-Ionized Water (Vol %)	Sodium Chloride (g/L)
Blood [38]	14%	86%	9.4

**Table 3:** Resonance frequencies of antennas when immersed in the bio-phantom.

Antenna	A	B
433 MHz	296 MHz	342 MHz
915 MHz	697 MHz	670 MHz

## Data Availability

Not applicable.
